# Cost-effectiveness thresholds or decision-making threshold: a novel perspective

**DOI:** 10.1186/s12962-023-00472-6

**Published:** 2023-10-03

**Authors:** Lihua Sun, Xiaochen Peng, Shiqi Li, Zhe Huang

**Affiliations:** 1 School of Business Administration, Shenyang Pharmaceutical University, Shenyang, Liaoning China; 2 Shanghai Health Development Research Center, Shanghai, 201199 China

**Keywords:** Pharmacoeconomic evaluation, Pharmacoeconomic threshold, Cost-effectiveness threshold, Decision-making, Reimbursements

## Abstract

The use of multiple cost-effectiveness thresholds in pharmacoeconomic evaluation is a hotly debated topic in the international academic community. This study analyzed and discussed thresholds in the context of pharmacoeconomic evaluation and reimbursement decision-making. We suggest that the thresholds inferred from reimbursement decisions should be distinguished from cost-effectiveness threshold in pharmacoeconomic evaluation. Pharmacoeconomic evaluations should adopt a fixed threshold, which should not vary with the subjects evaluated. This would help avoid the invitation of numerous cost-effectiveness thresholds for a specific drug, an exceptional disease, a type of innovation, or a certain level of malignancy, which misleads economic evaluation adopting restless changing standards and making pharmacoeconomic evaluation and decision-making more complex and contradictory.

## Introduction

Pharmacoeconomic evaluation (PE) are used in the pharmaceutical and health sectors in several countries, it provides a quantitative tool for reimbursement decisions. As a fast-developing academic discipline, besides conducting evaluations and generating results, many aspects of its theoretical basis are to be further explored. Cost-effectiveness analysis (CEA) is the most common approach used to investigate the incremental costs and health outcomes [typically expressed as quality-adjusted life-years (QALYs)] of two or more interventions. The conclusion of CEA is based on the incremental cost-effectiveness ratio (ICER) and its comparison to a threshold. Therefore, the cost-effectiveness threshold (CET) is inevitably a hot topic in academia and an unsolved problem in many countries [1]. There are three different approaches to estimate CET [2–4]: the willingness-to-pay (WTP), representative of welfare economics; the precedent method, based on the value of an already funded technology; and the opportunity cost method, which connects the threshold to the amount of health that is displaced. The setting of CET is influenced by many factors, most countries still adopt the CET of 1–3 times GDP per capita recommended by the World Health Organization (WHO) [5], while some countries have established their own thresholds, the UK is a representative country. National Institute for Health and Care Excellence (NICE) reports a maximum WTP threshold of £ 20,000–30,000/QALY (equivalent to 0.57–0.86 times GDP per capita in 2021 of UK) and allows for adjustment of the CET when factors such as social preferences and fairness are considered [6]. While Claxton et al. estimated the CET as £12,936 /QALY for the UK using the opportunity cost approach [7]. In China, Wang HY et al. reported the CET of about 0.63 times GDP per capita (2017) using the marginal cost method [8]; Wu J et al. calculated, through statistical life value method, that the CET was about 1.45 times of GDP per capita [9]. Ye ZP estimated the CET of $113,987/QALY (equivalent to 1.4 times China’s GDP per capita in 2021) based on WTP method [10].

In addition to research on methodology of threshold estimation, a crucial question is whether a fixed threshold or condition-based threshold should be used. A systematic review investigated 10 countries from 4 continents found that regions from 7 countries have two or more CETs [11]. Moreover, adjusting thresholds for different diseases and conditions appears to be a plausible solution to encourage innovation while ensuring the return on investment in research and development stage [12]. Furthermore, the values of ICER varied when calculated each ICER in each CEA using reimbursement price of each drug appraised. While the specification of CET in healthcare decisions and the approach of determining reimbursement price are not publicly stated in most countries. This study discusses this topic in the context of PE and decision-making, and provide our perspective.

## Main text

### Why a threshold matter

In the 1960s, the government was overwhelmed by the remarkable surge in health-care spending, in order to reasonably allocate limited resources, Weinstein and Zechauser first proposed the concept of “critical ratio” in the field of health resource allocation in 1973 [13]. The critical ratio is the critical point for resource allocation under budget constraints, where those with less than the critical ratio will receive resources and the opposite will not. This critical ratio is the prototype of the threshold.

In PE, the threshold is the decision rule to identify the optimal alternative by comparing the ICER with the predetermined threshold. In cost-benefit analysis (CBA) the threshold can be identified as “1” or the economic rate of return, for costs and benefits are both estimated in monetary terms [14]. However, in CEA and cost-utility analysis (CUA), an ICER in the southeast quadrant of the cost-effectiveness plane is rare, for achieving substantial incremental health benefits by little incremental costs requires revolutionary technological innovation. In most cases, a CET is required and indicates the monetary value of health outcomes for a specific country/region. Therefore, the abovementioned threshold is based on academic measurement and estimation, represented by the pharmacoeconomic threshold (PT) in this study.

Since problems such as continuing healthcare expenditure pressure, rising challenges from an aging population, expanding population with chronic conditions spread, CEA/CUA has become a feasible tool for efficient allocating limited healthcare resource. Thus, PT become a critical issue to primarily ensure the science on deciding healthcare resource allocation.

### Threshold in decision making

Decision-making is choosing an optimal course of action from all available alternatives to attain a goal (s) [15]. It starts with identifying the goals, followed by identifying the alternatives, choosing from among them, executing the relevant tasks, and reviewing till the goals are achieved. Decision-making could be for more than one objective or goal. The optimal choice for single-objective decision-making is straightforward. For example, if the PE is for single-objective decision-making, the cost-effectiveness alternative is the optimal choice. However, when decision-making is for multiple objectives, this optimal alternative cannot fulfill every objective or sub-objective. When decision-making goals escalate to the macro level, more dimensions and sub-objectives will be introduced to establish this overall goal [16]. Pharmaceutical Benefits Advisory Committee (PBAC) gives a special consideration for medicines meets “Rule of Rescue”, for example, which could reverse the decision that rejected the listing of this medicine due to not being cost-effective, by the same token, the decision-maker who accept “Fair-innings” argument may tend to prioritize the younger by adjusting the weight of the relevant sub-objective in the decisions. Thus, the sub-objectives and their weights can be adjusted as the overall goal, conditions, and decision-making environments change.

Some nations use the Health Technology Assessment (HTA) framework to evaluate new technology when making healthcare decisions. For instance, NICE’s HTA appraisal in the UK provide clinical guideline, economic evaluations, and social equity considerations. The results of the final review are directly applied to healthcare decisions [17, 18]; In certain countries, appraisal is separated from pricing and decision-making process. The clinical advantages and cost-effectiveness of pharmaceuticals are first evaluated by one organization, and pricing and decisions are then made by another organization depending on the findings of the appraisal and other factors. Although the opinions of a range of stakeholders are typically included in this process, the criteria for calculating prices and whether techniques like multi-criteria decision analysis (MCDA) are employed for decision-making are not made publicly available. For instance, in Canada, health technical assessments are carried out by Canada’s Drug and Health Technology Agency (CADTH) and/or Institute of Economic and Social Studies (INESS), while price negotiations and reimbursement decisions are made by pan-Canadian Pharmaceutical Alliance (pCPA). The final recommendation of CADTH and/or INESS is taken into consideration during the decision-making process, together with other elements like plan’s mandate, priorities, and resources [19]. Reimbursement decisions are fundamentally multi-objective problems, regardless of how economic evaluation impacts pricing and decisions. Even though the ICER can be inferred from the reimbursement price when data is openly accessible and available, this ICER value can only be used to understand past decisions and should not be regarded as an implicit threshold for economic evaluation because otherwise it will lead to the causal fallacy.

Even if both of the evaluated medications are novel, the inferred ICER thresholds are typically not the same values. The main cause of this is because different sub-objectives were given different weights while making decisions. Clearly, the sub-objective for economics should run concurrently with other sub-objectives, such as clinical benefits, for example, regardless of whether an HTA framework or decision science, such as MCDA, is utilized in decision-making. Therefore, the inferred ICER thresholds from decisions are not the thresholds in economic evaluation, and should not be adopted as the decision rule in economic evaluation.

### Thresholds in PE

PE helps make informed decisions regarding pharmaceuticals. In PE, there are three basic types of indicators: time, value, and efficiency/ratio indicators [14]. The cost-benefit analysis (CBA) method employs efficiency indicators, namely the incremental cost-benefit ratio(△B/△C), to ensure the efficient allocation of public sector resources in order to promote general welfare. In the context of the healthcare system, CBA is the first method employed in PE, and it uses efficiency indicator(△B/△C) that is completely consistent with them in the public sector, in which costs and benefits were quantified and measured in monetary form. According to the theory of welfare economic [20], Pareto optimality has been reached when a project’s incremental benefit exceeds its incremental cost, or when △B/△C ≥ 1, and the new project should be adopted for resource allocation optimisation. With a decision rule of “1,“ which indicates that incremental benefits and incremental costs are equal, CBA has an endogenous and distinctive threshold. The CEA/CUA in PE are similar to CBA in that they use efficiency/ratio metrics. However, the benefits of treatments and drugs related to health are challenging to be monetized. Therefore, effectiveness and utility have been invited to quantify the health outcome and benefits, while the effectiveness/utility and cost are measured in distinct units. As a result, there is a lack of an objective and endogenous threshold, as represented by “1” in the CBA. There are three basic techniques for estimating the CEA/CUA threshold: willingness to pay, precedent method, and opportunity cost method. The threshold derived by one of the aforementioned approaches in a particular scenario should, in theory, be a single, fixed value that performs as a function similar to ‘1’ in CBA. Although an alternative may be accepted when △B/△C is less than 1 in CBA, this does not imply that it is cost-benefit, the underlying reason is that more dimensions with higher weights are considered in the decision-making process. Based on this inference, the thresholds in CEA/CUA should be single and fixed values.

The global shared overall objective of pharmaceutical management is the realization of “Quality Use of Medicine”, [15] which is defined as: “patients receive medications appropriate to their clinical needs, in doses that meet their individual requirements for an adequate period, and at the lowest cost to them and their community.“ In other words, it ensures the safe, effective, cost-effective, and optimal use of medications, which is the primary objective of most decision-making in the healthcare system. Thus, PE should aim to serve one of the sub-objectives of “Quality Use of Medicine” with a single criterion. Although the “health outcome” should be inclusive and comprehensive in PE, the results and conclusion are limited to the economic and cost-effectiveness dimension. Similarly, the goal of developing an essential drug list and national reimbursement drug list comprises multiple sub-objectives. Economic is one of the multiple sub-objectives, which include selecting drugs with improved efficacy, safety, achieving societal equity, encouraging innovation and so on. The weights of these sub-objectives can change with the preference of stakeholders and societal conditions, which can be reflected in the decisions, however, that weights should not be used to change the threshold in economic evaluation. The PT should act as the leverage of weighing scale, and the measurement criteria should not change with the instrument. Therefore, the PT should be unified in evaluating different drugs/treatments for a specific objective.

## Conclusion and outlook

A PE provides quantitative evidence for decision-making related to health outcomes and costs in the pharmaceutical and health sector by supporting assessment of the “economic sub-objective”. Hence, the PT should be set to meet its sub-objective from a specific perspective in the process of decision-making. Moreover, in multi-objective decisions, the weight of each sub-objective depends on the decision maker’s preference, for example, decision-maker tends to weigh more on the economic dimension for common diseases, but less for life-threatening diseases. Therefore, the inferred thresholds from decisions are characterized by a set of condition-based values, which are derived from specific decisions under specific conditions. The weight of “economic sub-objective” in different decisions could change. However, as long as PE is used to conduct evaluation in order to fulfil the “economic sub-objective,“ whether the economic sub-objective is part of a multi-objective decision or as the sole objective in a single-objective decision, the PT should be a fixed value.

To sum up, rather than conditionally changing with the subjects, one fixed threshold should be referred to in the economic evaluation. Otherwise, it will result in two types of issues: (1) The fallacy of causation. More elements of other sub-objectives tend to be embedded to one sub-objective, leading to this one sub-objective being established as a surrogate for the overall goal, which confounds economic evaluation with decision science and complicates both domains. (2) Setting a specific threshold value for a distinctive subject or an exceptional condition may result in an expanding number of thresholds, which misleads economic evaluation adopting restless changing standards and ultimately makes it implausible to accomplish optimal allocation of healthcare resources.

Therefore, future work in this field should include: (a) identification of the overall goal of the decision-making and establish a set of sub-objectives that are mutually exclusive and collectively exhaustive, (b) research on the inclusion and exclusion criteria for selecting elements of value that fit the overall goal, and the framework to position them into proper sub-objectives, and (c) focus on new therapeutic areas such as rare diseases, for which patient populations are limited, and it is nearly impossible to recoup the investments at a low drug price. Multi-pathway solutions should be established, including tax incentives, academic funding, and financial instruments to encourage the development of new therapies. Nevertheless, the PT should be explicit and fixed when PE is employed as a tool to generate the conclusion under the economic sub-objective in scientific decision-making.

## Data Availability

Not applicable.

## Abbreviations

PE: Pharmacoeconomic evaluation
CEA: Cost-effectiveness analysis
CBA: Cost-benefit analysis
CUA: Cost-utility analysis
QALYs: Quality-adjusted life-years
ICER: Incremental cost-effectiveness ratio
CET: Cost-effectiveness threshold
WTP: The willingness-to-pay
WHO: World Health Organization
NICE: National Institute for Health and Care Excellence
HTA: Health Technology Assessment
PT: Pharmacoeconomic threshold
MCDA: Multi-criteria decision analysis
CADTH: Canada’s Drug and Health Technology Agency
INESS: Institute of Economic and Social Studies
pCPA: Pan-Canadian Pharmaceutical Alliance
